# Caryoscope: An Open Source Java application for viewing microarray data in a genomic context

**DOI:** 10.1186/1471-2105-5-151

**Published:** 2004-10-15

**Authors:** Ihab AB Awad, Christian A Rees, Tina Hernandez-Boussard, Catherine A Ball, Gavin Sherlock

**Affiliations:** 1 Dept. of Genetics, 300 Pasteur Drive, Stanford University Medical School, Stanford, CA 94305-5120, USA; 2 Dept. of Biochemistry, Stanford University School of Medicine, Stanford, CA 94305-5307, USA

## Abstract

**Background:**

Microarray-based comparative genome hybridization experiments generate data that can be mapped onto the genome. These data are interpreted more easily when represented graphically in a genomic context.

**Results:**

We have developed Caryoscope, which is an open source Java application for visualizing microarray data from array comparative genome hybridization experiments in a genomic context. Caryoscope can read General Feature Format files (GFF files), as well as comma- and tab-delimited files, that define the genomic positions of the microarray reporters for which data are obtained. The microarray data can be browsed using an interactive, zoomable interface, which helps users identify regions of chromosomal deletion or amplification. The graphical representation of the data can be exported in a number of graphic formats, including publication-quality formats such as PostScript.

**Conclusion:**

Caryoscope is a useful tool that can aid in the visualization, exploration and interpretation of microarray data in a genomic context.

## Background

The application of high-throughput technologies (such as DNA microarrays) to biomedical experimentation generates large quantities of data that can be difficult to browse and interpret in the absence of a graphical representation. Eisen *et al. *have previously displayed clustered microarray data using a false color representation that greatly aids in the intuitive interpretation of the data ([1]). However, when these data are from array comparative genome hybridization (arrayCGH) experiments (*e.g.*, see [2]), the genomic locations of the reporters (the molecules in each spot on a microarray) that were used to generate the data are important for interpretation. A relative increase or decrease in the ratios for a group of reporters that report on adjacent genomic locations may indicate amplification or deletion of that genomic region, respectively. Additionally, even the analysis of expression data in the context of genomic position can also identify regions of amplification or deletion, or even cases of aneuploidy ([3,4]).

In addition to being able to view and browse arrayCGH data, it is also important that the data be readily connected to annotation sources, such that a user can easily determine the identity and attributes of the gene represented by a reporter that was present on a microarray, which for instance may show evidence of amplification or deletion. For example, in arrayCGH experiments using tumor cells as the DNA source, there is an obvious value in rapidly determining whether a deleted region contains a tumor suppressor gene.

Finally, researchers frequently need to create figures, for publications, communication with co-workers, supplemental websites, or presentations. Thus researchers should be able to produce the visual representation of their data in a variety of graphic formats.

Caryoscope was originally implemented as a Web form, generating either a bitmap or a clickable PDF output. When this became an important day-to-day tool for our users ([5-9]), we created an improved, interactive version, consisting of a standalone application for analyzing arrayCGH data and an open architecture of re-usable classes that may be embedded by other developers in their own applications. In this paper, we focus on Caryoscope as an application.

Some other software packages were developed while this work was in progress, and can perform some of these functions. For instance, Genome2D ([10]) is designed to display bacterial transcriptome data on linear chromosome maps, while SeeCGH ([11]) was designed for viewing arrayCGH data (only for 2-channel arrays). However, both of these programs are designed to run solely on the Windows operating system, whereas Caryoscope is a Java application that can be used on Macintosh OS X, Linux and various UNIX operating systems, as well as Windows. Greshock *et al. *([12]) have built similar functionality, called CGHAnalyzer, on TIGR's Multiple Experiment Viewer (MeV) platform, but with a different (circular) whole-genome view. Furthermore, Caryoscope can be run in a command line mode, making it easy to embed within a CGI or a processing pipeline.

## Implementation

We implemented Caryoscope in Java ([13]) and deployed it as a Java Web Start ([14]) application, so a user may run it directly from our website ([15]) by clicking on a link. One can also install Caryoscope directly on a computer, but we recommend launching via the website in order to obtain the most current version of the software.

Caryoscope accepts data as text input files in simple formats so as to maximize interoperability with other systems.

## Results

### Application features

**Figure 1:**
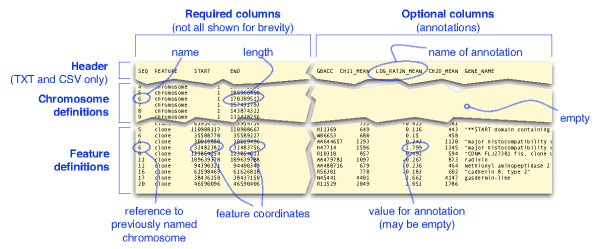
**The Caryoscope input file format. **An illustration of the input file format for Caryoscope. A tab-delimited (TXT) file is shown as an example, but the CSV and GFF formats are similar. Each column of data represents an annotation, with the name of the annotation at the top. Note how, at this level, there is no distinction between expression data and other information: for maximum flexibility, everything is an annotation.

As input, Caryoscope accepts a single file in either the General Feature Format (GFF, [16]) or a tab-delimited (TXT) or comma-delimited (CSV) spreadsheet-compatible format. This file describes the chromosomes to be displayed, and a set of loci on the chromosomes annotated by a number of associated microarray datasets and other descriptive information. The structure of a Caryoscope input file is illustrated in Figure 1.

**Figure 2:**
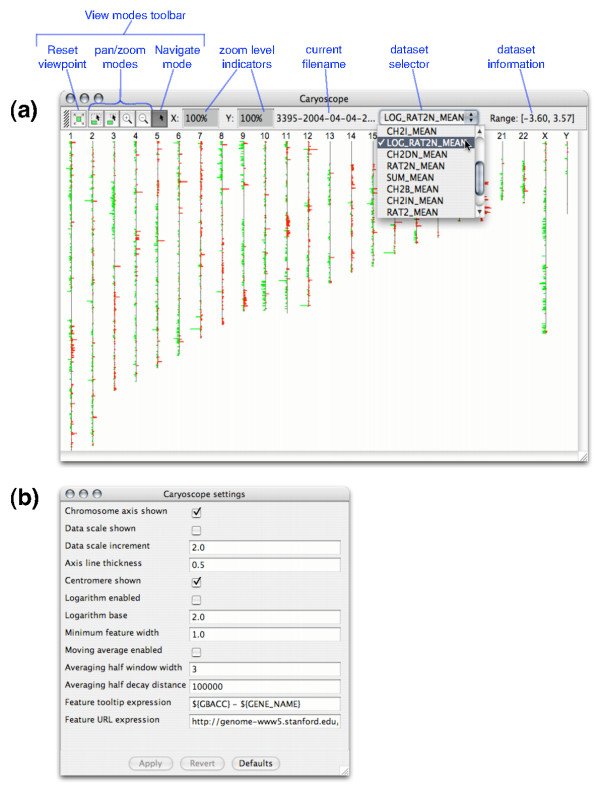
**The Caryoscope main window. **(a) Caryoscope displays all numerical annotations from the data file as datasets. The datasets are available for viewing via the dataset selector at the top of the Caryoscope window. (The data shown are provided by Jon Pollack, from a breast tumor sample. Note the significant regions of amplification and deletion.) The toolbar at the top allows the user to select a variety of modes for viewing the data. The panning and zooming modes allow the user to navigate the data, while the **Reset viewpoint **button returns to display all of the data in the current dataset, scaled to the current window size. The **Navigate **mode enables hyperlinking and tooltips. The zoom level indicators improve the user's situational awareness while viewing the data. (b) The **Settings **window allows the user to configure the application.

Once the user opens a file, Caryoscope automatically displays one of the datasets contained therein (Figure 2). Caryoscope displays each feature as a rectangle on the chromosome axis; the size of the rectangle on the horizontal axis, perpendicular to the chromosome, represents the magnitude of the associated data value, while the size of the rectangle in the vertical direction, along the chromosome axis, represents the size of the represented feature, based on its genomic coordinates. Pursuant to convention, the default display of Caryoscope represents positive values in red bars, which are drawn to the right of the chromosome, and negative values in green bars (though these colors can be changed), which are drawn to the left of the chromosome. Thus, based on color, size, and location, researchers can easily intuit the meaning of the graphical representation of their data.

Caryoscope provides several modes in which the user may view the data; these are controlled by the **View modes **toolbar (Figure 2). In the various panning and zooming modes, the user may change the view of the data to drill down to specific regions of interest. In **Navigate **mode, the user sees tooltips (small informational pop-up windows) that appear immediately when mousing over the features, and can navigate to related URLs by clicking on each feature. Typically, users at Stanford University (our primary source of testers and users) link GenBank accessions, associated with the cDNA clones that are on their microarrays, to SOURCE Gene Pages ([17]).

**Figure 3:**
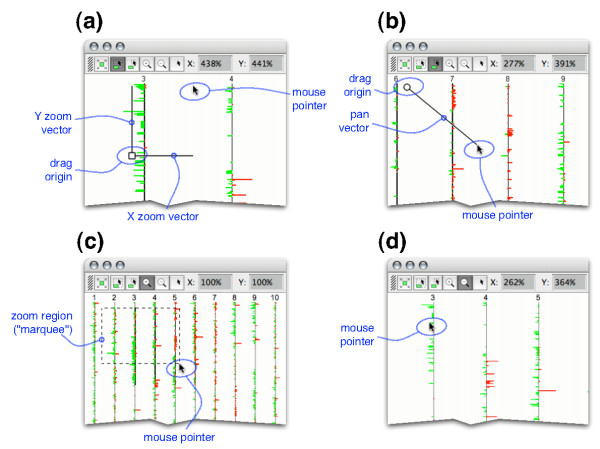
**Zooming and panning modes. **The zooming and panning modes are selected from the **View modes **toolbar. (a) In **Dynamic zoom **mode, the user clicks then drags the mouse; based on the mouse motion, the view zooms continuously with independent zoom speeds in the X and Y directions. (b) In **Dynamic pan **mode, the user clicks then drags the mouse; the direction and distance of the mouse motion determines the speed and direction of the panning motion through the data. (c) In **Zoom in **mode, the user selects a region (denoted by a "marquee"). The display is zoomed so that the selected region is fit to the current window. The user may also click anywhere within the current window, which zooms in by a fixed scaling, centered on the click point. (d) In **Zoom out **mode, the user clicks anywhere, which zooms out by a fixed scaling, centered on the click point.

The zooming paradigm in Caryoscope is somewhat novel in that it permits independent control of the zoom scales in the X and Y directions (Figure 3). It allows users to select the best scaling to see detail along the chromosome axis, and the data values perpendicular to the chromosome axis, for their specific data. The **Reset viewpoint **button on the **View modes **toolbar (Figure 2) allows the user to return quickly to the default scaling.

**Figure 4:**
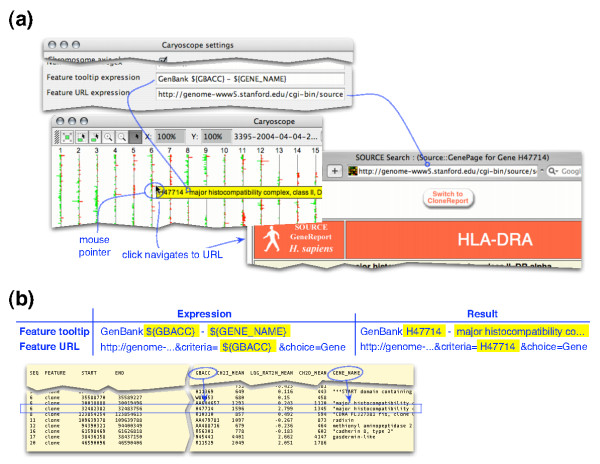
**Navigate mode. **(a) In this mode, Caryoscope shows a tooltip (small informational pop-up window) whenever the user moves the mouse over a feature. When the user clicks on the feature, Caryoscope navigates to a URL related to the feature. Both the tooltip text and the URL are generated dynamically from the annotations on the feature under the mouse pointer, via the **Feature tooltip expression **and **Feature URL expression **settings. (b) Illustration of how the tooltips and URLs are computed from the **Feature tooltip expression **and **Feature URL expression **settings and the annotations in the input file.

The behavior of Caryoscope in **Navigate **mode is shown in Figure 4. The tooltip and URL text are computed for each feature by substituting the value of its annotations into the **Feature tooltip expression **and **Feature URL expression **settings, as illustrated in the figure. These features allow users to have immediate access to information about each feature as they browse the data.

The user can enable two built-in computations on the data values: a user can compute the logarithm of the values (to any base specified by the user), and a user can compute a moving average of the values. Both these computations can be controlled from the **Settings **dialog (see Figure 2b). Users can perform other computations outside Caryoscope; this is facilitated by the fact that we support common spreadsheet-compatible file formats (TXT and CSV).

To prepare diagrams, the user can export the Caryoscope display to a variety of graphics formats via the **Export **dialog. Specifically, Caryoscope supports vector (*e.g.*, PostScript and PDF) output for scalable publication-quality results, and raster (*e.g.*, JPEG and PNG) output for ease of viewing, posting on supplemental websites, and inclusion in presentations.

A user may export graphics from Caryoscope via the command line mode, without having to invoke the interactive user interface. For example, to export a view of a dataset as a PDF file, the user could invoke Caryoscope as follows:

      java -jar caryoscope-run.jar

           -inputFile 3395-2004-04-04.csv

           -visibleDataset RAT2_MEAN

           -outputFile 3395.pdf

           -outputFormat pdf

All settings may be modified via the command line. The -listFormats option provides a list of available graphics output formats, and the -help option prints a brief summary of the options.

A comparison of the features of Caryoscope with other, similar software is presented in Table 1.

### Obtaining Caryoscope

In addition to immediately executable copies, the complete source code for Caryoscope is available without limitations from our website ([15]), and is covered by a very liberal Open Source ([18]) license (the MIT License, [19]). All external components used by Caryoscope are also Open Source.

We update Caryoscope frequently (approximately once every three weeks) and post news items on the website. We also send e-mail announcements to people who have requested them.

## Discussion

### Biological context

Caryoscope is useful for viewing both arrayCGH and expression data in the context of genomic position. It helps a biologist gain insight by providing a high-level view of a large amount of data at once, where patterns can be perceived at a glance.

A biologist studying amplifications or deletions in tumor cells may create and export graphics representing arrayCGH and expression data for the same cells using Caryoscope, and visually compare the two side-by-side. For instance, co-located regions that are amplified at the DNA level and over-expressed at the RNA level would provide excellent confirmation of the results. Using the zooming and panning features of Caryoscope, the biologist could focus on specific regions of interest.

**Figure 5:**
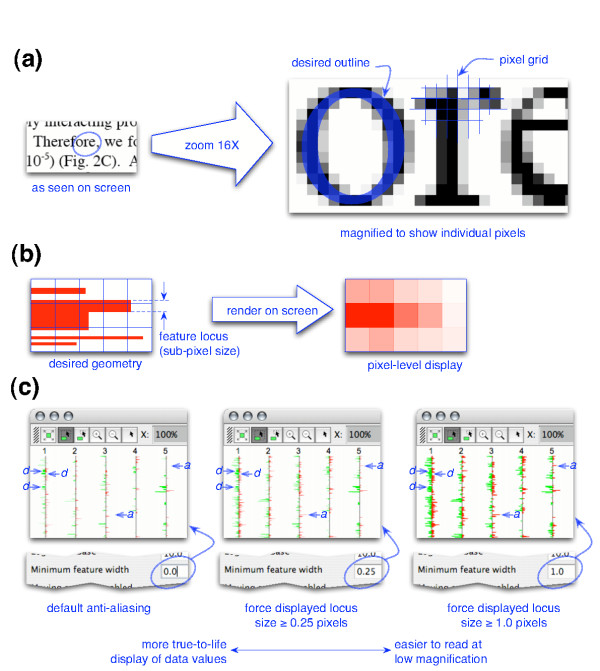
**Example of anti-aliasing. **A demonstration of the effect of anti-aliasing in Caryoscope. (a) Anti-aliased text displayed by Adobe Acrobat 6.0 on an Apple computer running Macintosh OS 10.3.3. Note that the text appears as if drawn by smooth black ink but, upon magnification, it becomes clear that this smoothness is a visual illusion created by careful manipulation of the color of each pixel. (b) The analogous situation in Caryoscope, where the scaled sizes of the feature loci are smaller than one pixel on the display device. In this example, we applied a form of anti-aliasing by *prefiltering*, where we computed the color of each pixel based on the area of red rectangles it contains. See [20]. (c) Actual displays from Caryoscope showing how the **Minimum feature width **setting highlights different aspects of the data. The three deletions ***d ***on chromosome 1 are clearly distinct at a setting of zero, but are lost in the midst of other artificially expanded features when the setting is increased. On the other hand, the two amplification "spikes" ***a ***on chromosomes 3 and 5 are invisible at a setting of zero, but become visible when the setting is increased.

To identify regions of aneuploidy, the biologist can again simply examine the data visually. In this case, however, one would look for a large-scale pattern. One might specify a **Minimum feature width **of, say, 2 pixels (Figure 5c), to ensure that any deletion or amplification, no matter how small in genomic coordinates, is easily visible. Rather than zooming in on specific regions, a researcher would tend to compare overall views of the entire genome. If it seems like practically all of one chromosome is amplified or deleted, the biologist would have strong evidence for aneuploidy.

In a gene expression study, a biologist may suspect that some expression patterns are correlated with genomic position. Caryoscope allows one to view expression data, either for the whole genome or on a region of interest, to help confirm or refute a hypothesis.

Finally, in all this work, the biologist may want to have quick and easy access to information about the genomic features displayed. As long as the information needed is available from the annotations that were saved in the input data file (or available at a URL that can be built based on the annotations), one can use the **Feature URL expression **and **Feature tooltip expression **(Figure 4) settings to provide immediate mouse-over feedback with this information – almost as if the application were customized for a specific field of interest.

### Software context

We intend Caryoscope to be a bench-top visualization tool that biologists can use immediately to get day-to-day work done, with a very low "cost of entry" for getting started. This led us to a number of design choices.

Caryoscope can be launched from our web site without a prior installation step. Since it is Open Source, anyone, including any for-profit organization, can use it without restrictions and without having to obtain a license or register for access.

The input file formats and output graphical formats we chose are all in common usage. In particular, the TXT and CSV input formats can be generated using any popular spreadsheet or database software, or even with a plain text editor, without having to do any programming.

Finally, we built Caryoscope to be content-neutral, with no hard-coded specificity to any research field. Thus, users of Caryoscope may control how annotations are treated as numerical data, and can "program" data-driven interactive behavior of the display (*i.e.*, the tooltips and hyperlinks).

### Future work

From the outset of this project, our biggest challenge has been how to accurately represent the huge amounts of data in a typical gene expression or arrayCGH experiment using the limited number of pixels available on the screen or on a printed page. If we skew our display algorithms too much towards producing a "sharp", high-contrast plot, we risk obscuring detail in the data and leading biologists to the wrong conclusions. On the other hand, since the size of the data elements, properly scaled from genomic coordinates to the display device, can be far less than the size of one pixel, we need a supportable way to "summarize" the data within each pixel and represent that summary as a single value: the color (including the brightness) of that pixel.

Modern computer graphics systems use a technique called "anti-aliasing" ([20] and Figure 5a) to render sub-pixel details with the illusion of smoothness. The Java subsystems we use in Caryoscope do this automatically and, in the current version of the software, we simply rely on them (Figure 5b). However, the anti-aliasing in Java is designed to display visually appealing text, lines and arcs, but not to ensure the most accurate possible on-screen rendering of scientific data. Specifically, at low magnification, the data almost disappear unless we force a minimum pixel size for each locus (Figure 5c).

We will develop our display methods further to ensure that we can provide an easy-to-read display while retaining the subtle variations in the data. Following the spirit of medical diagnostic imaging (DICOM, [21]), whereby incorrect details in a few pixels could lead to an incorrect conclusion, we must ensure that our displays, which are used for important research decisions, are never misleading.

One solution, suggested by [12], is to display the data elements, not aligned to the position of the loci along the chromosome, but rather in strict sequential order with a fixed width. While this solves the anti-aliasing problems, it does eliminate consistent chromosomal positions and alignments of the data. Furthermore, it causes the appearance of the display to be dependent on the specific choice of clones – which can be another source of subtle variation when comparing multiple datasets. Another idea is to display dots, rather than horizontal bars, so that the "spread" of the data is more visible even if data points are super-imposed. We will investigate this for a future release of Caryoscope.

We are particularly concerned about the use of Caryoscope (or similar) graphics in vector formats (such as PostScript, PDF and SVG) that are subsequently rendered on diverse display devices and printers. Since we have no control over the rendering at the destination, it is likely that the same vector output could look very different on different devices. Once we have studied this problem in more detail, we intend to provide practical usage guidelines for researchers.

**Figure 6:**
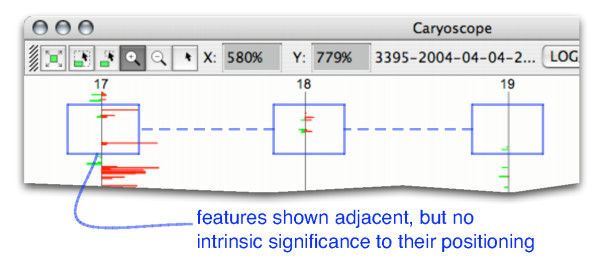
**Zooming paradigm. **An example of a partially zoomed-in display, which highlights adjacent regions of four chromosomes. The model of continuous 2-dimensional zooming breaks down in this case, since the adjacency of these regions is not biologically relevant.

Our experience with the application, and how it is used, leads us to believe that the current zooming paradigm should be revisited. While the model of a continuously zoomable 2D space provides users with the features they need, it can lead to displays whereby the data in specific regions of adjacent chromosomes are juxtaposed, even though their being next to each other is not intrinsically meaningful (see Figure 6). (An exception to this might occur in telomere amplification, or perhaps special behavior around the centromeres. In this case, the ability to align the chromosomes at either end, or at the centromeres, would be helpful.)

In the future, we will modify our display so that the user can turn "on" or "off" the display of the available chromosomes. Within that display, and with the help of our users, we will review the role of the X-axis scaling: perhaps it should change the scaling of the data, or perhaps it does not belong in Caryoscope-like applications at all.

A common user request is to display two or more arrayCGH and/or expression datasets side by side, either to determine regions of recurrent deletion or amplification, or to discern visually the impact that changes in chromosome copy number may have on transcript levels (*e.g.*, see [5]). Another frequent request is to show the cytoband information for each chromosome. We intend to add these features as part of our future development, in the course of which we would perhaps redefine, or extend, the manner in which our input data are defined (*i.e.*, we would accept the definition of the chromosome names, lengths and cytobands in a separate file that would be re-used by different datasets).

### Summary

Caryoscope currently provides a flexible method to visualize, explore and create images of microarray data in a genomic context. With such a tool, microarray researchers will be able to answer questions about how genome copy number or genome position plays a role in biological processes or human diseases.

## Conclusions

Caryoscope is a useful, flexible Java application for the visualization of microarray data in a genomic context. It is available as Open Source under the permissive MIT License, allowing anyone to use or modify it.

## Availability and requirements

**Project name: **Caryoscope

**Project home page: **

**Operating system(s): **Platform independent

**Programming language: **Java

**Other requirements: **Java 1.4 or higher

**License: **MIT License

**Any restrictions to use by non-academics: **None

## Authors' contributions

IABA compiled the functional requirements for the Java version of Caryoscope and designed and implemented the software. CAR conceived and wrote the original Perl version of Caryoscope. THB aided and participated in the design of the study, dealt with user support and worked with users to get feedback and suggestions for further development. CAB aided and participated in the design and evaluation of the study and provided feedback and suggestions for future development. GS supervised and participated in the design of the study. All authors read and approved the final version of the manuscript.

## Figures and Tables

**Table 1:** Comparison to other tools Itemized comparison of the features of Caryoscope ([15]), CGHAnalyzer ([12],[22]) and SeeCGH ([11],[23]).

	**Caryoscope**	**CGHAnalyzer**	**SeeGH**
**Platform**	Java (runs on any popular platform)	Java (runs on any popular platform)	Microsoft Windows
**Application availability**	Unlimited use; no signup	Unlimited use with signup	Free with signup for academics
**Installation process**	Install Java; run application directly from Web	Install Java; install application	Install MySQL; install application; configure database tables
**Input file formats**	Single input file; spreadsheet compatible text or General Feature Format (GFF)	Multi-component input; text-based input formats	Text-based input formats
**Internal data format**	Text file	Text file or database	MySQL database
**Source code availability**	Free download	Download with signup	Not available
**Source code license**	Open Source	Unknown	N/A
**Internal code dependencies**	All Open Source	Depends on some commercial software	N/A
**Superimposed karyotype plot**	No	Yes	Yes
**Informational summary for each feature**	Yes, configurable in application settings	Yes, fixed tabular format	Yes, fixed tabular format
**Web link from each feature**	Yes, configurable in application settings	Yes, fixed Ensembl or GoldenPath links	No
**Data points in genome coordinates**	Yes	Yes	Yes
**Data points in linear order**	No	Yes	No
**Simultaneous display of multiple experiments**	No	Yes	No
**Zoomable display**	Yes	No	Yes
**Part of a more complete analysis framework**	No	Yes (TIGR MeV)	No
**Built-in data point filtering**	No	No	Yes
**Whole-genome view**	Linear chromosomes	Concentric circles representing chromosomes	Linear chromosomes
**Generate graphics from command line**	Yes	No	No
